# N-doped carbon dots covalently functionalized with pillar[5]arenes for Fe^3+^ sensing

**DOI:** 10.3762/bjoc.15.123

**Published:** 2019-06-07

**Authors:** Jia Gao, Ming-Xue Wu, Dihua Dai, Zhi Cai, Yue Wang, Wenhui Fang, Yan Wang, Ying-Wei Yang

**Affiliations:** 1 State Key Laboratory of Inorganic Synthesis and Preparative Chemistry, International Joint Research Laboratory of Nano-Micro Architecture Chemistry (NMAC), College of Chemistry, Jilin University, 2699 Qianjin Street, Changchun 130012, P. R. China; 2 International Joint Research Center for Nanophotonics and Biophotonics, School of Science, Changchun University of Science and Technology, 7089 Satellite Road, Changchun 130022, China; 3 The State Key Laboratory of Refractories and Metallurgy, School of Chemistry & Chemical Engineering, Wuhan University of Science and Technology, Wuhan 430081, P. R. China

**Keywords:** chemical sensor, CN-dots, fluorescence, ion recognition, supramolecular chemistry

## Abstract

Fluorescent N-doped carbon dots (CN-dots) covalently functionalized with carboxylatopillar[5]arene (CP[5]), namely CCDs, have been prepared the first time. Compared with CN-dots without pillarene units, the newly constructed ﬂuorescent CCDs could recognize Fe^3+^ with high selectivity. Therefore, such CCDs can potentially serve as a promising chemical sensor for Fe^3+^ ions.

## Findings

Carbon dots (C-dots) as a rising star in fluorescent nanomaterials have attracted wide attention in terms of preparation and application [1–4]. Fluorescent C-dots possess advantages over organic dyes and traditional semiconductor quantum dots due to their high solubility, facile modification, desirable chemical stability, and high resistance to photobleaching. Thus, fluorescent C-dots have been widely applied in the fields of imaging, catalysis, and sensing [5–7]. With the rapid development of fluorescent C-dots, much effort has been made to tune their electrical, optical, and chemical properties via doping and surface modification to offer novel C-dots with attracting properties [8]. It is worth noting that surface modification can not only tune the functional groups of fluorescent C-dots but also endow a variety of functionalities to C-dots and expand the applicability of C-dots [8–10].

Synthetic macrocyclic compounds, especially new macrocyclic arenes, have become one of the research hotspots in supramolecular chemistry [11–14]. Among them pillarenes as a relatively new family of pillar-shaped members discovered a decade ago have played a key role due to their unique conformations, symmetrical structures, superior host–guest properties, supramolecular assembly characteristics, and versatile functionalities [11,15–16]. Carboxylatopillar[5]arene (CP[5]) is one of the most popular functional pillarenes that has been exploited in many research areas, especially in sensing and detection [16–21]. To our best knowledge, only few reports on pillarene-modified C-dots can be found in the literature [19], not to mention their potential applications in ion sensing.

**Scheme 1:**
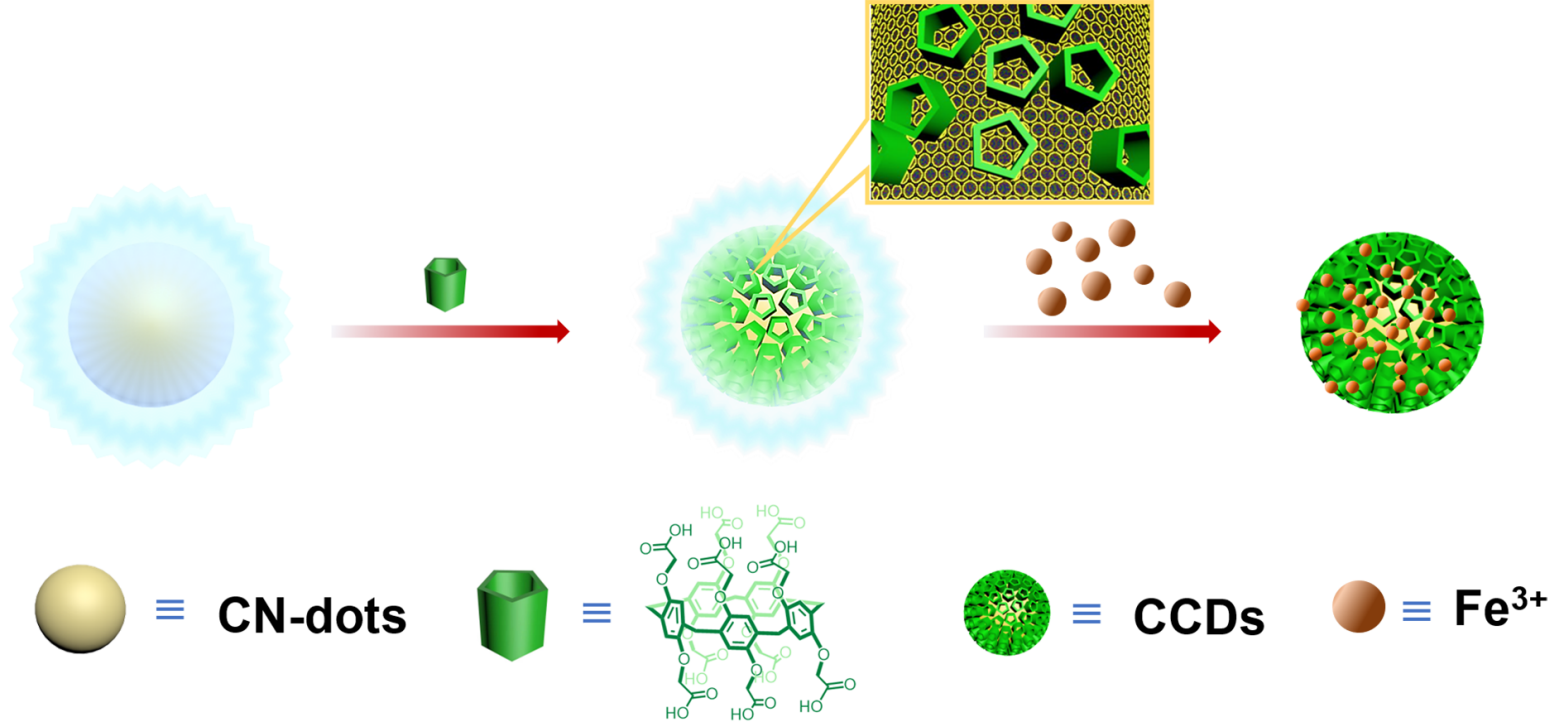
Schematic illustration of the synthesis of CCDs and its use for Fe^3+^ sensing.

Herein, we prepare fluorescent C-dots, namely CCDs, by linking various CP[5] rings on N-doped C-dots (CN-dots) through covalent bonds formed by EDC-NHS coupling reaction. The comparison of CCDs with single CN-dots without CP[5], revealed that the CCDs exhibit a higher selectivity for Fe^3+^ detection (Scheme 1). Fe^3+^ ions play an important role in the living organism and is related to many diseases, thus it is vital to develop effective Fe^3+^ sensing materials [7,22–24]. Our current strategy suggests that CCDs with excellent selectivity for Fe^3+^ sensing represent a new type of advanced fluorescent sensing materials.

**Figure 1:**
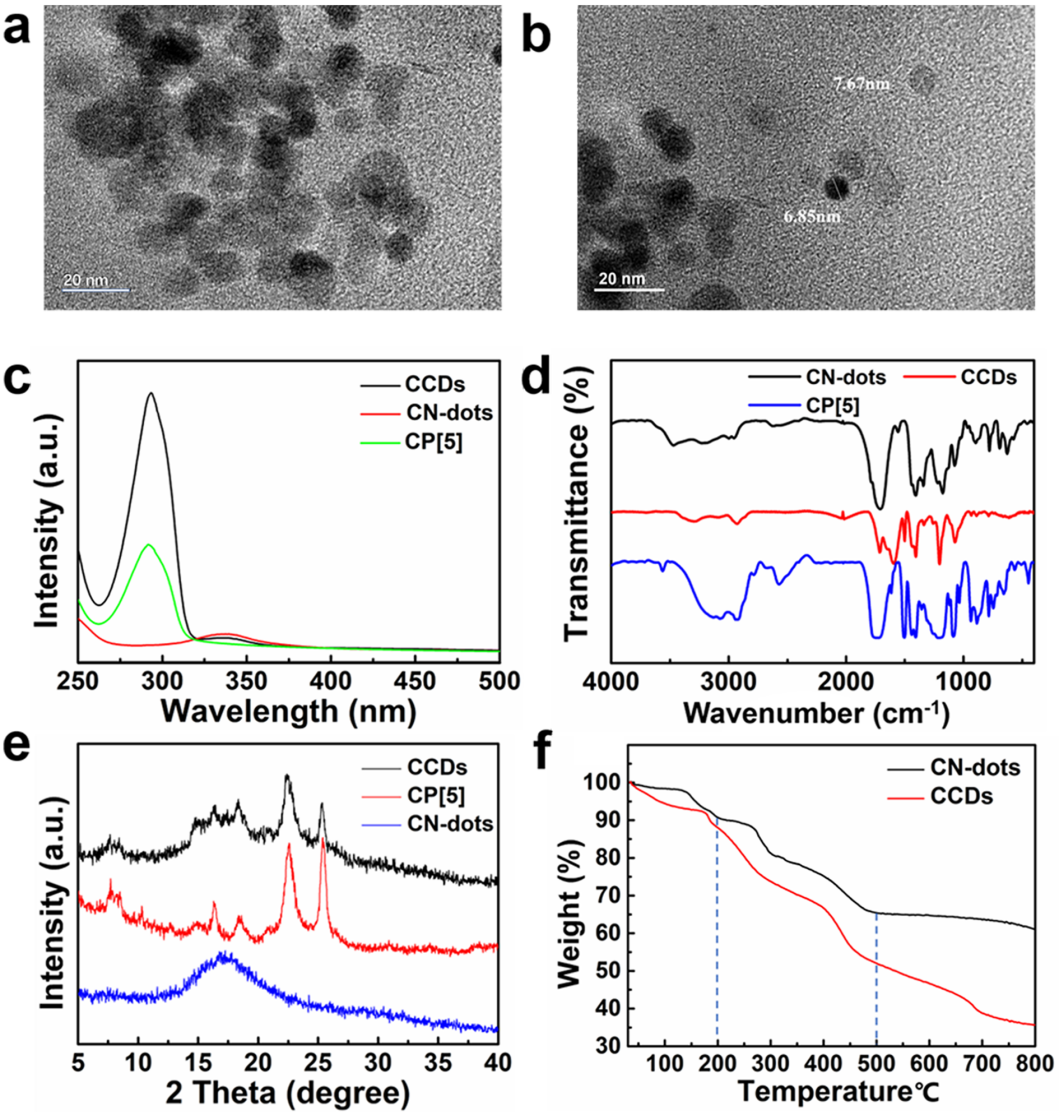
TEM images of a) CCDs and b) CN-dots. c) UV–vis spectra of CP5, CN-dots, and CCDs. d) FTIR spectra of CP5, CN-dots, and CCDs. e) PXRD patterns of CP5, CN-dots, and CCDs. f) TGA curves of CN-dots and CCDs.

The sizes and morphologies of CCDs and CN-dots were characterized by transmission electron microscopy (TEM) (Figure 1a). TEM images showed the spherical shape of CCDs with a diameter of 9 nm. For comparison, the shape of CN-dots was quasi-spherical with an average diameter of 7 nm (Figure 1b).

As shown in Figure 1c, the UV–vis absorption peak of CN-dots is at 340 nm, and CP[5] has an obvious UV–vis absorption peak at 295 nm. As expected, the UV–vis spectra of CCDs show two absorption peaks, one at 295 nm and the other at 340 nm, indicating that CP[5] has been linked on the surface of C-dots. The Fourier transform infrared spectra (FTIR) of C-dots, CN-dots, and CP[5] are shown in Figure 1d. Firstly the surface of the CN-dots was investigated, the bands from 760 to 910 cm^−1^ were attributed to the C–H bending vibration, and the sharp absorptions at 1708, 1406, and 1176 cm^−1^ were assigned to the stretching vibration of –C=O, asymmetric stretching vibrations of C–N and the wagging vibration of –NH_2_, respectively. In addition, the broad band at ca. 3100 cm^−1^ can be attributed to the stretching vibrations of –NH_2_ and –OH bonds. As for CP[5], several characteristic absorption bands could be observed, where the broad band at 3100 cm^−1^, the strong band at 1725 cm^−1^, the sharp absorptions at 1502 and 1442 cm^−1^, and the peaks from 650 to 910 cm^−1^ were assigned to stretching vibrations of –OH bonds and –C=O group, the bending vibration of –OH, and the asymmetric stretching vibrations of C–O and C–H of benzene rings, respectively. Compared with CN-dots, the relative intensity of the asymmetric stretching vibration of C–N at 1433 cm^−1^ in CCDs was obviously increased because the abundant amino groups of CN-dots reacted with carboxylic acid groups of the CP[5] macrocycles.

Powder X-ray diffraction (PXRD) patterns (Figure 1e) of CCDs show the characteristic peaks of CN-dots and CP[5], further confirming the successful synthesis of CCDs. Thermogravimetric analysis (TGA, Figure 1f) revealed that the weight loss at 200 °C was due to the pyrolysis of the amino and oxygen groups on the surface of CN-dots, and upon reaching the temperature of 500 °C, CN-dots began to decompose and 61 wt % of its weight was lost until 800 °C. Meanwhile, the weight loss of CCDs reached ca. 35 wt %, indicating that ca. 26 wt % of CP[5] macrocycles were covalently grafted on the surface of CN-dots [17,19].

**Figure 2:**
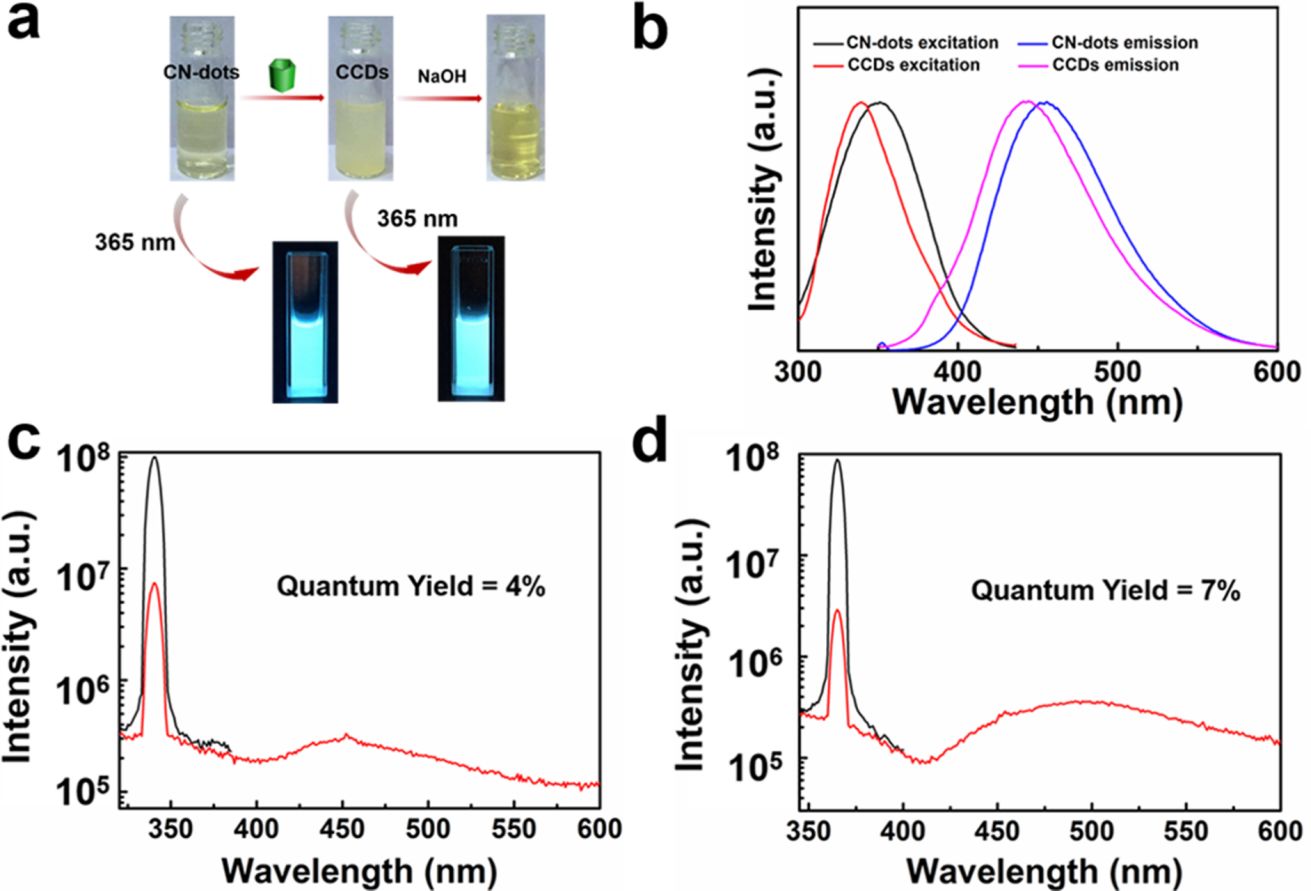
a) Photographs of CN-dots and CCDs in aqueous media in natural light, and under excitation with a UV lamp (365 nm). b) Emission (blue line) and excitation (black line) spectra of the CN-dots in water, and emission (pink line) and excitation (red line) spectra of the CCDs in water. Quantum yields of c) CCDs and d) CN-dots. Experimental conditions: Slit widths: Ex. 5 nm, Em. 5 nm.

As shown in Figure 2a, the as-synthesized CN-dots material is soluble in water while the CCDs solid is insoluble. When NaOH was added slowly into the suspension of CCDs, the turbid system became clear, indicating that the CP[5] was successfully anchored on the surface of CN-dots. More intuitively, both the CCDs and the purified CN-dots emit blue fluorescence under the excitation at 365 nm, further illustrating that the CCDs retain the luminous property of CN-dots.

Emission and excitation spectra of CCDs and CN-dots in aqueous solutions are provided in Figure 2b. The maximum emission and excitation wavelength of CCDs are 445 nm and 340 nm, and the maximum emission and excitation wavelength of CN-dots are 475 nm and 365 nm, which coincides with the blue light emission. The obvious blue shift was due to the modification of CP[5], which led to the increase of n–π* transition energy with the formation of new amide bonds [4,25]. The PL quantum yield (QY) of the CCDs aqueous solution was determined to be 4% (Figure 2c), while the CN-dots solution exhibited stronger emission intensity with 7% QY (Figure 2d).

**Figure 3:**
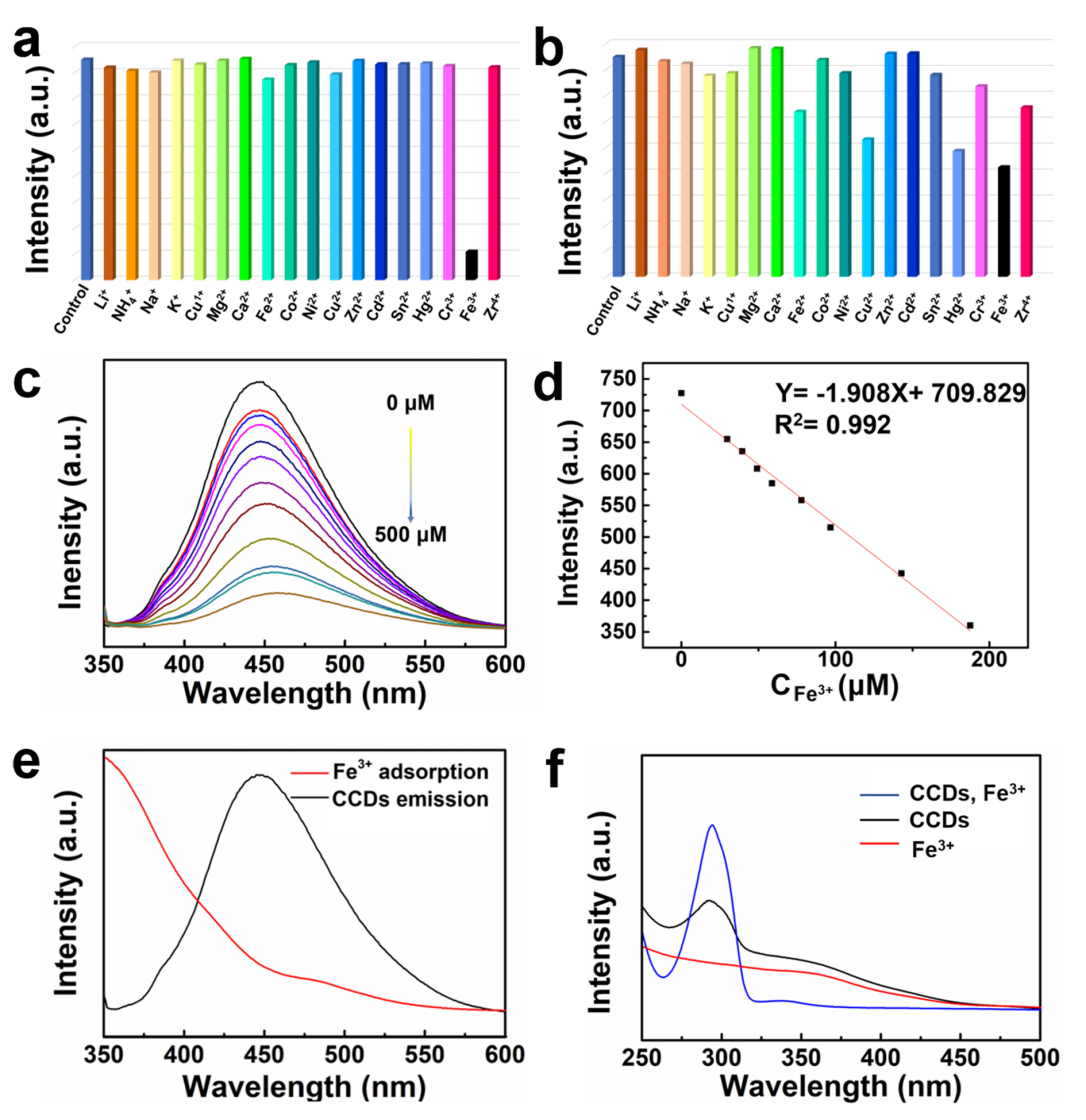
Fluorescence quenching degrees of a) CCDs and b) CN-dots in the presence of different metal ions. The concentrations of metal ions were 400 μМ. c) Fluorescence spectra of a CCDs system in the presence of different concentrations of Fe^3+^ ions (0–500 μM). d) Linear curve of the relative fluorescence intensity versus the concentrations of Fe^3+^ ions. Experimental conditions: Slit widths: Ex. 5 nm, Em. 5 nm; λ_ex_ = 340 nm. e) Spectral overlap of the UV–vis absorption spectrum of Fe^3+^ (red line) and fluorescence spectrum of CCDs (black line). f) UV–vis spectra of CCDs, Fe^3+^, and CCDs after the sensing of Fe^3+^.

To investigate the application of the as-synthesized fluorescent CCDs in ion sensing, various chlorides of metal ions (Li^+^, NH_4_^+^, Na^+^, K^+^, Cu^+^, Mg^2+^, Ca^2+^, Fe^2+^, Co^2+^, Ni^2+^, Cu^2+^, Zn^2+^, Cd^2+^, Sn^2+^, Hg^2+^, Cr^3+^, Fe^3+^, and Zr^4+^) were added into the fluorescent CCDs system, respectively. As shown in Figure 3a, Fe^3+^ could almost completely quench the fluorescence of CCDs among all metal ions at a final concentration of 400 μM, illustrating that CCDs could serve as a potential fluorescence probe for identifying Fe^3+^. Figure 3b shows the fluorescence intensity change of CN-dots in the presence of metal ions. The experimental results suggested that there are several metals ions that could simultaneously quench the fluorescence of CN-dots, further indicating that CCDs is superior to CN-dots in the selective sensing of Fe^3+^. In addition, the fluorescence intensity of CCDs decreased gradually with the stepwise addition of Fe^3+^ in the range of 0–500 μM under the excitation at 340 nm (Figure 3c). As illustrated in Figure 3d, the fluorescence intensity versus the concentration of Fe^3+^ ions (0–190 μM) established a good linear correlation (R^2^ = 0.992) and the lowest detection limit (LOD) was determined to be 1.2 μM [16,24–25].

Subsequently, the quenching mechanism of Fe^3+^ toward CCDs was investigated. As demonstrated in Figure 3e, the UV–vis absorption of Fe^3+^ overlaid with the emission peak of CCDs, thus, quenching performance can be contributed to the energy transfer between ions and the materials [26]. Meanwhile, the UV–vis absorption peaks of CCDs exhibited no shift and regeneration before and after Fe^3+^ sensing, indicating that the fluorescence quenching is not resulted from the formation of new composites, and the highly selective sensing towards Fe^3+^ is also related to the binding of Fe^3+^ with CP[5] ring (Figure 3f) [24,27–28].

## Conclusion

In summary, a promising fluorescent chemical sensing material (CCDs) was synthesized for the first time by covalently attaching CP[5] macrocycles onto the surface of CN-dots via EDC-NHS coupling reaction. Compared with unmodified CN-dots, CCDs demonstrated novel properties after the introduction of CP[5], and showed a higher selective sensing ability for Fe^3+^ ions. Hopefully, this strategy will inspire the design of new fluorescent chemical sensors toward Fe^3+^ ions with high selectivity.

## Supporting Information

File 1 Experimental section.
