# Supplementary material for: N-doped carbon dots covalently functionalized with pillar[5]arenes for Fe3+ sensing
Source: Beilstein J Org Chem. 2019 Jun 7;15:1262–7. doi: 10.3762/bjoc.15.123 (PMC6604737; doi:10.3762/bjoc.15.123)
Supplement: File 1 — Experimental section. [file Beilstein_J_Org_Chem-15-1262-s001.pdf]

BEILSTEIN JOURNAL OF ORGANIC CHEMISTRY

Supporting Information

for

N-doped carbon dots covalently functionalized with pillar[5]arenes for Fe^{3+} sensing

Jia Gao, Ming-Xue Wu, Dihua Dai, Zhi Cai, Yue Wang, Wenhui Fang, Yan Wang and Ying-Wei Yang

Beilstein J. Org. Chem. **2019,** *15,* 1262–1267. doi:10.3762/bjoc.15.123

Experimental section

License and Terms: This is a supporting information file under the terms of the Creative Commons Attribution License (http://creativecommons.org/licenses/by/4.0). Please note that the reuse, redistribution and reproduction in particular requires that the authors and source are credited.
The license is subject to the *Beilstein Journal of Organic Chemistry* terms and conditions: (https://www.beilstein-journals.org/bjoc)

Experimental section

Materials

Citric acid, ethylenediamine, *N*-hydroxysuccinimide (NHS), and 1-ethyl-3-(3-dimethylaminopropyl)carbodimide hydrochloride (EDC) were purchased from Aladdin. Carboxylatopillar[5]arene (CP[5]) was synthesized according to our published procedure [S1]. N-Doped carbon dots (CN-dots) covalently functionalized with CP[5] rings, namely CCDs, were prepared from CN-dots via an EDC-NHS coupling reaction with CP[5] macrocycles [S2]. All the chemicals used in our experiments were of analytical grade and used as received without further purification. Deionized water was used in all relevant experiments.

Preparation of CN-dots

CN-dots were prepared according to the reported method [S3]. Briefly, ethylenediamine (100 μL) and citric acid (3 g) were dissolved in water (10 mL) and the solution was heated at 160 °C for 6 h. During the reaction period, the reaction solution changed from colorless to brown, suggesting the formation of CN-dots. Finally, the CN-dots were obtained by dialysis and lyophilization.

Preparation of CP[5]-functionalized CN-dots (CCDs)

The CP[5] molecules were linked through the $–NH_2$ groups located on the surface of the CN-dots by an EDC-NHS coupling reaction. CP[5] (10 mg) was dispersed in deionized water (10 mL) and the mixture was activated for 1 h, then, EDC (60 mg), NHS (100 mg), and CP[5] (10 mg) were added. The reaction mixture was stirred at room temperature for 24 h. Finally, the product was dialyzed for 3 days at room

temperature to remove the impurities.

Characterization

Transmission electron microscopy (TEM) images were obtained on a JEM 2100F instrument at an accelerating voltage of 200 kV. Fourier transform infrared (FTIR) spectra were recorded on a Bruker Vertex 80 V spectrometer. Powder X-ray diffraction (PXRD) measurements were carried out by using a PANalytical B.V. Empyrean powder diffractometer. Ultraviolet visible (UV–vis) spectra were recorded on a Shimadzu UV-2550 instrument. Thermogravimetric analysis (TGA) was done on a Q500 instrument. Fluorescence spectra were recorded in quartz cuvettes on a Shimadzu RF-5301PC spectrophotometer.

References

S1. Li, H.; Chen, D. X.; Sun, Y. L.; Zheng, Y. B.; Tan, L. L.; Weiss, P. S.; Yang, Y. W. *J. Am. Chem. Soc.* **2013,** *135*, 1570-1576.

S2. Zhou, T.; Yu, H.; Liu, M. Q.; Yang, Y. W. *Chin. J. Chem.*, **2015,** *33*, 125-130.

S3. Zhu, S.; Meng, Q.; Wang, L.; Zhang, J.; Song, Y.; Jin, H.; Zhang, K.; Sun, H.; Wang, H.; Yang, B. *Angew. Chem., Int. Ed.*, **2013,** *52*, 3953-3957.
